# Living With Hypochondroplasia: A Qualitative Exploration of Children's and Caregivers' Experiences, Challenges, and Unmet Needs

**DOI:** 10.1002/mgg3.70151

**Published:** 2025-11-14

**Authors:** Elisabeth M. Oehrlein, Reni Pekala, Stacie Cavallaro, Margaret Cho, Chandler Crews, Andrew Dauber, Ankita Saxena, Joe Vandigo, Emily S. Reese

**Affiliations:** ^1^ Applied Patient Experience, LLC Washington DC USA; ^2^ Hypochondroplasia Families Community Greystones Ireland; ^3^ BioMarin Pharmaceutical Inc. Novato California USA; ^4^ The Chandler Project Little Rock Arkansas USA; ^5^ Children's National Hospital Washington DC USA

**Keywords:** hypochondroplasia, patient‐centered research, patient‐focused drug development, qualitative research, skeletal dysplasia

## Abstract

**Background:**

Hypochondroplasia (HCH) is a rare genetic skeletal dysplasia characterized by short stature, disproportionate limbs, and complications such as learning differences. Currently, no treatments are approved to address HCH‐related short stature, which can adversely affect quality of life. This study aimed to explore diagnostic processes, care pathways, daily life impacts, and unmet needs in HCH.

**Methods:**

Ninety‐minute interviews were conducted with nine children and young adults and 25 caregivers who had physician‐confirmed HCH. Participants discussed diagnostic journeys, treatment considerations, and day‐to‐day challenges. Following interviews, two 90‐min focus groups among caregivers (*n* = 10) were conducted to explore themes emerging during interviews.

**Results:**

We found that diagnostic pathways vary significantly, with signs of HCH identified in utero or during infancy or early childhood. Families described complex psychosocial burdens that include impacts on daily activities and emotional challenges due to height differences and disproportionate limb length. Additionally, many people with HCH have complications that go beyond short stature and include developmental delays, learning differences, and seizures. Families desire more support and resources related to HCH.

**Conclusion:**

Future efforts should focus on holistic, patient‐centered strategies to better support individuals with HCH and their families.

## Graphical abstract

Summary of findings from a qualitative study on the experiences and unmet needs of children with hypochondroplasia and their caregivers.

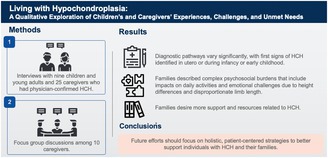

## 1 Background

Hypochondroplasia (HCH) (HCH; OMIM #146000) is a rare genetic skeletal dysplasia. Distinguishing features include short stature and disproportionate arms or legs. Although these features are generally less pronounced in HCH than in achondroplasia (OMIM #100800), learning differences and seizures may occur more frequently in HCH than in the general pediatric population (Legare and Basel 2023; Bober et al. 2020; Linnankivi et al. 2012). Prevalence estimates for HCH range from approximately 1 in 15,000–1 in 40,000 worldwide, affecting individuals across diverse ethnic backgrounds (Bober et al. 2020; Stevenson et al. 2012). Diagnosis typically involves identifying characteristic physical features and X‐ray findings, with genetic testing confirming changes in the *FGFR3* (fibroblast growth factor receptor 3; HGNC:3689; OMIM 134934) (Bober et al. 2020). Children with HCH often experience complications such as epilepsy and learning difficulties that are typically managed through specialty care (e.g., neurology, occupational therapy, or physical therapy) (Bober et al. 2020).

No precision treatments are currently approved for HCH and care is focused on symptomatic management. Therapeutic decisions are driven by parental goals and concerns related to their child's growth (Dauber et al. 2024; Bober et al. 2020). Growth hormone therapy is considered experimental because studies have shown mixed clinical effectiveness (Pinto et al. 2024; Massart et al. 2015). Limb‐lengthening surgery can increase overall height, yet it is often considered controversial due to its highly invasive nature and prolonged discomfort (Bober et al. 2020; Lie and Chow 2009; Paley 2021). Vosoritide, a medication recently approved for achondroplasia, is now under investigation for HCH (Dauber et al. 2024).

Emerging evidence suggests that children and adults with short stature, regardless of etiology, may experience lower overall quality of life (Backeljauw et al. 2021). One cross‐sectional survey in Japan, involving mainly individuals with achondroplasia and some with HCH, outlined the physical, mental, and social accommodations undertaken to address their challenges (Ajimi et al. 2022). A structured questionnaire demonstrated impaired quality of life in children with (Dauber et al. 2024). However, additional knowledge is needed to fully understand the day‐to‐day experiences of those affected by skeletal dysplasia, including HCH (Moy et al. 2023). To our knowledge, no qualitative studies to date have explored the perspectives and experiences of children with HCH and their caregivers.

The goal of this study was to gain a more in‐depth understanding of the experiences of children with HCH and their families, focusing on the diagnostic process, care pathways, and the impact of short stature and other complications on daily life. Another objective was to explore current and potential treatments for HCH, emphasizing key unmet needs and patient‐centered outcomes.

## 2 Methods

### 2.1 Ethical Compliance

This study received approval from Advarra's institutional review board (Pro00075366) and was conducted according to the ethical principles outlined in the 1964 Helsinki Declaration. Informed consent was obtained from caregivers, with assent provided by children and adolescents before their participation in interviews and focus groups.

### 2.2 Eligibility and Recruitment

Participants were recruited through social media and referrals from healthcare providers. We required evidence of a physician‐confirmed HCH diagnosis (e.g., physician notes, health records, or genetic test results). Individuals were invited to take part in the study after confirming that they met the inclusion criteria (see Table 1).

**TABLE 1:** Eligibility criteria.

Inclusion criteria	Exclusion criteria
Diagnosed with hypochondroplasia or child diagnosed with hypochondroplasia (ages 8–18 years) Ability to participate in an interview lasting approximately 90 min (about 1 and a half hours) Comfortable reading and communicating in English Currently live in the United States Access to the internet either through a computer or tablet	Blindness or significant visual impairment that would hinder their ability to see Currently incarcerated

### 2.3 Data Collection

Semi‐structured, 90‐min virtual interviews were conducted with children diagnosed with HCH and caregivers of a child with HCH. An interview guide based on the National Health Council's Patient Experience Mapping Toolbox was modified to ensure relevance to this study population (Oehrlein et al. 2024). All study materials were reviewed by a child living with HCH and their parent. Interviews took place between December 2023 and March 2024 via Zoom (Zoom Video Communications Inc.). Participants could choose whether to remain on or off camera for the duration of the interview. Participants could take breaks as needed and were asked midway if they wanted one. Sample interview questions can be found in the Supporting Information.

Subsequently, two focus groups were conducted in September 2024 with caregivers of children and adolescents who have HCH to explore topics that emerged during the interviews or the literature (Billich et al. 2023). These included experiences with specific HCH‐related complications: learning difficulties, developmental delays (e.g., speech and motor milestones), mental health challenges (anxiety, depression), sleep apnea, epilepsy or seizures, joint pain, recurrent ear infections, hearing loss, and weight management. Participants were asked to discuss the relative impact of each complication. Sample focus group questions appear in the Supporting Information.

### 2.4 Data Collection and Analysis

All interviews and focus groups were audio‐recorded, transcribed verbatim, and analyzed thematically using line‐by‐line inductive coding in ATLAS.ti. Two researchers independently coded the first two transcripts from each population (children and caregivers), then reviewed and compared codes to ensure consistency before coding the remaining transcripts. Two qualitative researchers also independently coded each focus group transcript. A thematic analysis was conducted. This study is reported in alignment with Standards for Reporting Qualitative Research (SRQR) (O'Brien et al. 2014).

GenBank (RefSeq) reference for FGFR3: NM_000142.5 (MANE Select); genomic reference: NC_000004.12 (GRCh38).

## 3 Results

### 3.1 Participants

Table 2 summarizes the demographic characteristics of those who participated in the interviews. Nine children and 25 caregivers (parents or grandparents) were interviewed, representing 25 unique children. Eight child‐caregiver dyads were interviewed separately, and one dyad was interviewed together. The children with HCH were nearly equally divided between female (52%) and male (48%) participants, with most identifying as non‐Hispanic (88%) and 68% identifying as White.

**TABLE 2:** Interview participant characteristics, data from dyads only counted once to reflect the characteristics of the child with HCH.

Characteristic	*n*	(%)
Child age today, years (mean [min–max])		10.1 [4–23]
Child age today, category		
4–7 years	6	24%
8–12 years	10	40%
13–16 years	8	32%
17–18 years	0	—
> 18 years	1	4%
Child age at HCH diagnosis		
Infancy (birth to < 1 year)	17	68%
Toddler (≥ 1 year to < 3 years)	7	28%
Child (≥ 3) years	1	4%
*Z*‐score of height of children with HCH at interview		
< −2.0	24	96%
≥ −2.0	1	4%
Sex at birth		
Female	13	52%
Male	12	48%
Ethnicity		
Hispanic	3	12%
Non‐Hispanic	22	88%
Race		
American Indian or Alaska Native	0	—
Asian	0	—
African American	1	4%
Native Hawaiian or Other Pacific Islander	2	8%
White	17	68%
Two or more races	5	20%
Other	0	—
Community setting		
Rural	5	16%
Suburban	16	64%
Urban	4	5%
Census region		
Midwest	6	24%
Northeast	3	12%
South	8	32%
West	8	32%
Household income		
< $25,000	2	8%
$25,000–49,000	0	—
$50,000–99,000	4	16%
$100,000–149,000	8	32%
≥ $150,000	11	44%
Complications and comorbidities^a^		
Shorter than peers	25	100%
Shorter legs and/or arms	24	96%
Enlarged head size	18	72%
Bowlegs	14	56%
Learning difficulties/cognitive impairment^b^	11	44%
Limited elbow extension	10	40%
Recurrent ear infection	10	40%
Motor development delays	8	32%
Obstructive sleep apnea	7	28%
Pain (joints, back, or neck)	7	28%
Epilepsy or other seizure disorder	5	20%
Anomalies on brain MRI	4	16%
Hearing loss	4	16%
Lumbar lordosis	4	16%
Central sleep apnea	2	8%
Kyphosis (rounding of the upper back)	2	8%
Scoliosis	2	8%
Hydrocephalus	0	—
Neuropathy	0	—
HCH treatments received (if any)		
Growth hormone therapy	6	24%
Limb lengthening surgery	6	24%

^a^ Participants were able to select no or multiple complications and comorbidities, or treatments.

^b^ For example: language delay, ADHD, autism, trouble concentrating.

All children with HCH were reported to be shorter than their peers (100%), and the majority also had shorter limbs (96%) or larger head sizes (72%). Other frequent complications included bowed legs (56%), learning difficulties or cognitive impairments (44%), limited elbow extension (40%), recurrent ear infections (40%), motor delays (32%), and joint, back, or neck pain (28%). Six children (24%) had taken growth hormone therapy, and another six (24%) had undergone limb‐lengthening surgery.

After the interviews, we re‐contacted a convenience sample of twelve parents who had expressed interest in participating in future research. Out of these, eleven parents responded and agreed to participate in focus groups. Ultimately, ten parents from ten unique families took part in the study. Additional details regarding participant characteristics in this subset are available in Table S1.

### 3.2 Interview Results

#### 3.2.1 Journey to Diagnosis

##### 3.2.1.1 Interviews

The first signs and symptoms of HCH can appear during pregnancy or early childhood. Some families learn of HCH in pregnancy due to ultrasound findings of short long bones or halted bone growth. At birth or soon after, signs such as disproportionate limb length, an enlarged head, pauses in breathing, or seizures may be apparent. In infancy, slowed growth or bowed legs may prompt further investigation. Among toddlers or young children, missed developmental milestones—particularly speech or motor skills—can lead caregivers to suspect an underlying issue.

**FIGURE 1:**
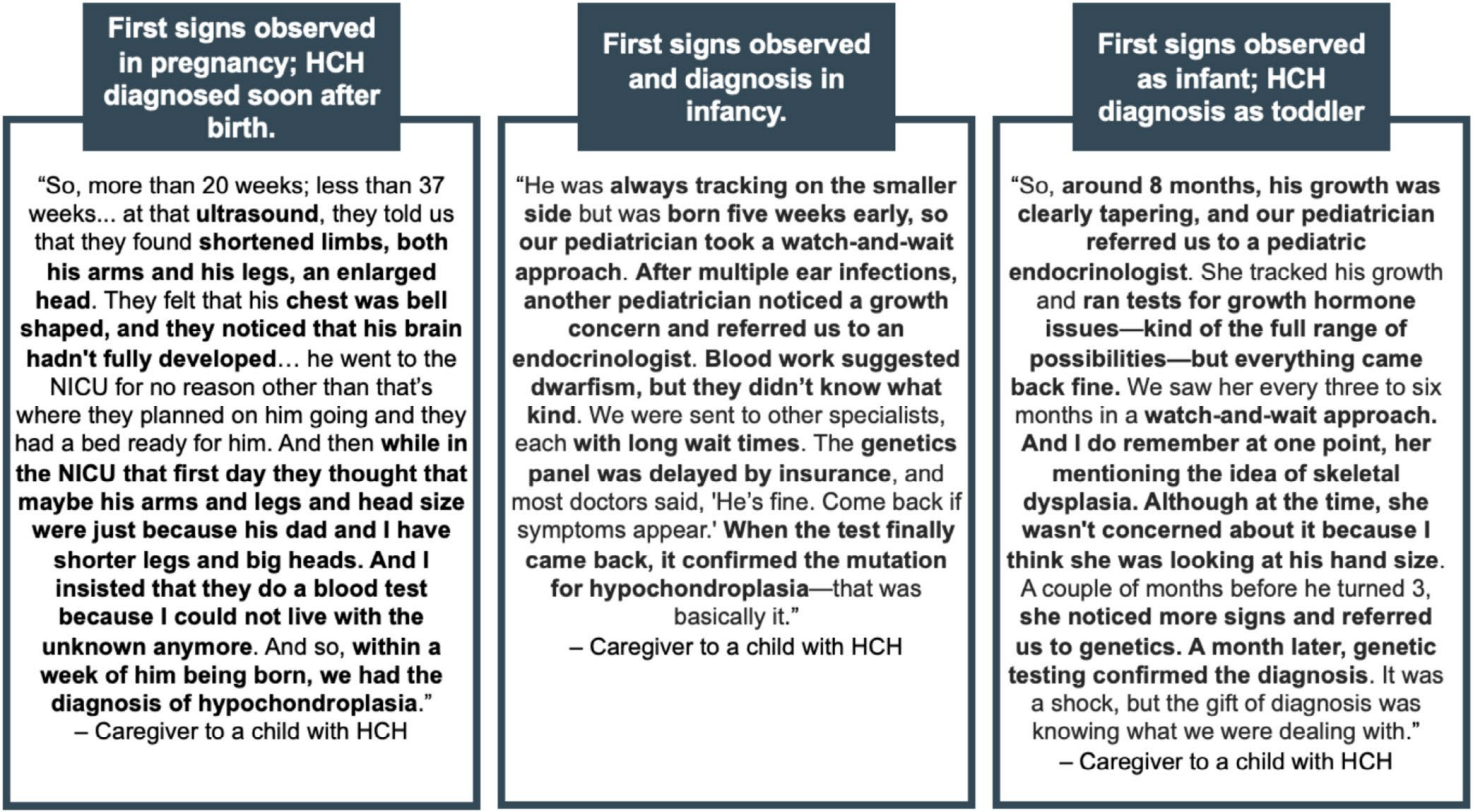
First signs and symptoms of HCH.

There is no single diagnostic pathway for HCH (see Figure 1 for vignettes). When signs arise during pregnancy or shortly after birth, diagnosis tends to be more direct; however, observing signs later often requires a chance encounter with a specialist familiar with skeletal dysplasia. Clinical and radiological assessments are typically conducted, followed by genetic testing to confirm the diagnosis.

Participants described several factors that can delay diagnosis. These include extended wait times—four to six months—for specialist appointments and up to two months for test results. Delays often occur once patients enter the healthcare system because providers may not suspect skeletal dysplasia, particularly if the obvious features of achondroplasia are not present. Additionally, a child's shorter stature may be mistakenly attributed to family traits, and misinterpretations of X‐rays or other findings can further hinder an accurate diagnosis.

I think it was a lot of that people don't know what hypochondroplasia is. Our pediatricians had never heard of it. And so, if you don't know, or if you're just not very familiar with the types of dwarfism, then it's easy to put everyone in one bucket and be like, “Dwarfism, it looks like this.” And obviously, we know that is not the case. There's so many different types of dwarfism. —Caregiver to a child with HCH

Many caregivers were initially surprised by their child's diagnosis and struggled to find reliable information on HCH. Several mothers recounted feeling guilty or worrying they had contributed to their child's HCH during pregnancy. Over time, most expressed relief that HCH was not life‐threatening but remained concerned about the implications of HCH on daily life. Most families deferred telling their children about the diagnosis until late childhood or the teenage years, when children themselves became more aware of size differences. Upon learning about HCH, these young people voiced concerns about longevity and the potential impact on their personal goals.

When I was a kid, it didn't really occur that I was smaller, but then as I got to fifth, sixth grade, I started noticing I'm smaller than a lot of these kids. So, then my parents knew I was onto something. Then they said, “We're gonna talk to you,” and then they told me that I had hypo and they explained it all to me. —Child with HCH

##### 3.2.1.2 Focus Groups

Focus group participants highlighted difficulties finding healthcare professionals (HCPs) experienced in caring for people with skeletal dysplasias, including HCH. Parents reported that in many cases, their children's doctors were unfamiliar with HCH, leading to delays in diagnosis. They recommended targeted education and making genetic testing more accessible and easier to navigate. Simplifying access to testing and ensuring that healthcare providers are aware of these options could help families get more rapid and more accurate diagnoses.

#### 3.2.2 Care After Diagnosis & Experiences With Available Treatments

##### 3.2.2.1 Interviews

After diagnosis, most children receive care from a primary care physician or geneticist, with various specialists—such as ENT physicians—engaged as needed to address specific complications. Less than half of the participants pursued limb‐lengthening surgery, growth hormone therapy, or both. Two took part in clinical trials after being referred by specialists on their care team. Six children underwent limb lengthening, which was described as painful and logistically difficult but ultimately beneficial because it led to an increase in height and limb length, which helped the child to complete daily tasks.

It's been amazing. She's still the shortest kid in her class. But it's just little things around the house that she can do now that she couldn't do before as far as reaching things, accessing things … She can almost touch the floor now when she's sitting in chairs, or at least a lot of chairs she can. We don't have to hem all of her pants anymore. —Caregiver of a child with HCH who received limb‐lengthening surgery

Six children received growth hormone therapy. Five of six caregivers were unsure whether growth hormone therapy was effective. They reported no side effects, and one attributed it to muscle growth, while another thought it might be responsible for one or two inches. One caregiver felt their expectations for increased height with growth hormone therapy had not been managed appropriately during the decision‐making process.

He gets injections every day for that. We haven't really seen any drastic improvement in his growth. But it seems, though, to be working marginally, if I might use that term … he might have gotten an inch or two, but nothing of what I would say is really significant, what we're looking for. —Caregiver of a child with HCH taking growth hormone therapy

While some caregivers and children were still considering different treatment options, other participants preferred not to pursue invasive or disruptive treatments, such as surgery, or those not approved for HCH, such as growth hormone therapy. Several participants stated that their child's endocrinologist or geneticist had discussed growth hormone therapy with them, but they had advised that it might not significantly increase their child's height.

I [would] have to basically skip a summer or be in a wheelchair at school. I'd have to skip a lot of sports too because I play sports basically year‐round. —Child with HCH describing why they are not interested in limb lengthening surgery

##### 3.2.2.2 Focus Groups

Focus group participants described a lack of access to knowledgeable HCPs and feeling uncertain about what steps to take after their child's HCH diagnosis. They expressed a strong need for clearer guidance on the implications of HCH, what to expect in the long term, and which HCPs their child should see. They emphasized the need for better education about HCH in the medical community to improve care and support for affected families.

Probably the hardest part of the whole experience was the not knowing what it meant. And I made the mistake of getting on Facebook pages where people were talking about worst‐case scenario things, and I think that really frightened me. And then the more and more that we had appointments and the more and more things that got checked, the more doctors were like, “Well, he's okay.” We'll see you in a year. —Parent of a child with HCH

#### 3.2.3 Meaningful Treatment Benefits

##### 3.2.3.1 Interviews

When asked about desired treatment outcomes, most participants referenced increased height and/or improved body proportions (Table 3). They believed that greater height or lengthened limbs would aid with routine activities—such as showering, using the bathroom, and reaching shelves—while also reducing the psychosocial impacts of short stature.

I think I want to be taller… Just an average height. Like how old I am. —Child with HCH

Make myself grow taller like I'm supposed to. —Child with HCH

**TABLE 3:** Examples of meaningful treatment benefits.

Increased height	Proportionate or longer limb length	Maintain physical functioning and independence	Increase height and limb length, as well as HCH complications
“I do think having those extra inches makes a big difference in your life … anything you can do to help your child have more access and success in the world, and not just aesthetically, just in terms of being able to reach the shower to turn it on. Or just all of these very practical things of navigating the world can make a huge difference in his life.” —Parent of a child with HCH	“Reach is almost a bigger deal than the height. People just automatically think height because she's short. But having the short bone in your arm, she could have a stepstool in the science lab at school to wash her hands. So, then now she can reach the sink because she has the stepstool, but she still can't reach the soap dispenser that's on the wall.” —Parent of child with HCH	“I would say how I function, just like making sure that I'm healthy enough to go about life pretty much the same as anybody else would, minus the fact that I'm shorter than most people are and may need to adapt in certain ways.” —Child with HCH	“It would make her long bone longer. So, making her taller. But like I said, not just taller, the arms is huge. Beyond that, it would be to find some sort of magic solution to her behavioral inattentive, distractibility, reading comprehension, anxiety.” —Parent of child with HCH

Other meaningful treatment benefits described by participants, typically in tandem with increasing height or short stature, include long‐term independence, an increased ability to stand for long periods of time, maintaining physical functioning over time, and decreased risk of spinal injury, seizures, and learning disabilities. Several participants stated they wished the underlying genetic causes of HCH could be fixed.

##### 3.2.3.2 Focus Groups

Focus group participants also reiterated that short stature and disproportionate limb length are distinct complications. Differences in height and limb length can lead to feelings of self‐consciousness and limit children's ability to participate in age‐appropriate activities. Participants stated that increasing limb length would significantly improve their children's physical abilities and confidence.

He wishes he had longer arms. Apparently, he's totally okay with his legs. I hem all of his pants for him. He's learning how to sew because he wants to learn how to hem his own pants. But he would like to have longer arms. —Parent of a child with HCH

One focus group participant also stated that reduced inflammation would be a meaningful treatment benefit.

#### 3.2.4 Daily Life With HCH & Unmet Needs

##### 3.2.4.1 Interviews

###### 3.2.4.1.1 Short Stature and Disproportionate Limb Length

Short stature and disproportionate limb length have a strong negative impact on daily life and social participation. Caregivers also described challenges related to their limb length, which can make everyday tasks more difficult. For example, one caregiver shared that their child struggles to reach items on high shelves or use standard‐sized furniture. Other caregivers described their child's difficulty using the bathroom or washing hands independently due to limb length. Some children were unable to participate in attractions at theme parks; for example, their height was not tall enough for rollercoasters.

He still has issues with wiping his own bottom because of the limb size. Issues, as maybe rides, like going to the fair or an amusement park, where age‐wise he should be able to ride, he can't. —Caregiver of a child with HCH

###### 3.2.4.1.2 Emotional Impacts

Many children with HCH face emotional challenges, especially regarding insecurities about their height and bullying at school. Caregivers, particularly of boys, mentioned societal expectations related to height and the importance of boosting self‐confidence. Some adolescents worried about whether they would be able to drive, while others felt uneasy when they were stared at in public settings.

I know what they're staring at but that's my weak spot. If I see you stare, automatically I assume it's because of my height and I automatically get upset … It's self‐conscious. —Child with HCH

###### 3.2.4.1.3 Accommodations and Modifications

**FIGURE 2:**
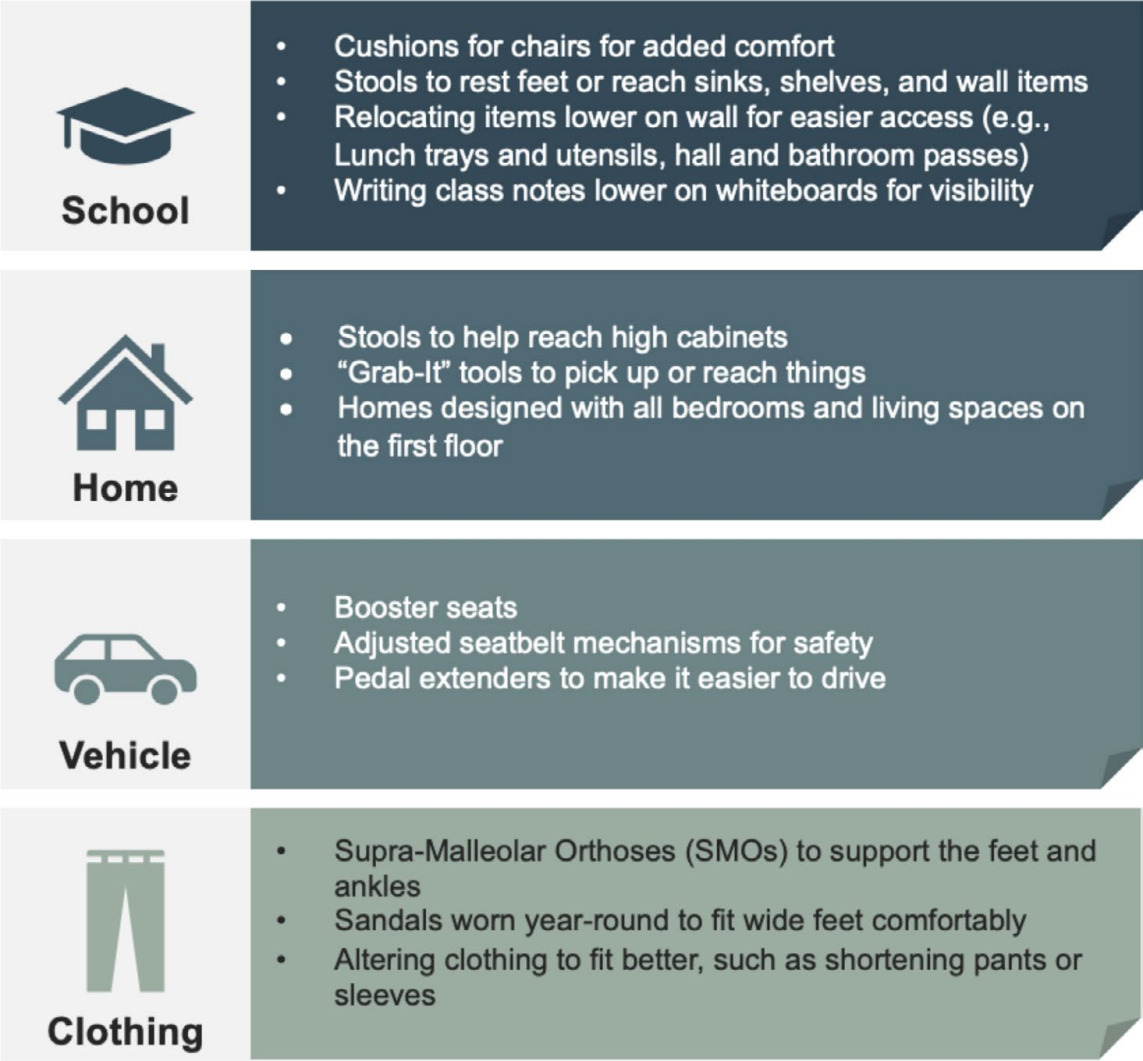
Examples of accommodations to account for short stature and reach.

A variety of adaptations helped children navigate their environments more comfortably (see Figure 2). At school, stools and adjustable seating were common, along with lowered items in classrooms. Prolonged booster seat use in cars, as well as pedal extenders and car adaptations, provided added independence. Clothing modifications were frequently necessary, with families seeking extra‐short sizes or learning to tailor garments themselves.

Because I'm not a sewer, he cannot wear jeans and stuff like other kids. He wears athletic shorts that fit like pants. And that's what he wears year‐round because that's what's comfortable. Even long‐sleeved shirts, he can't wear long‐sleeved shirts because they cover his hands, and he don't like them rolled up. —Caregiver of a child with HCH

###### 3.2.4.1.4 Sports, Hobbies, and Public Spaces

Caregivers noted that as children got older, differences in size became more pronounced, making sports dependent on height or size, such as basketball or hockey, impractical or high risk for injury. Some families sought alternatives in non‐contact sports such as swimming, gymnastics, or climbing, with many children excelling at these sports. Caregivers also noted that sporting equipment can be problematic. For instance, helmets and swim goggles often fail to fit correctly due to differing head proportions. One caregiver added that dance shoes do not fit well because of different foot proportions. In public spaces, poorly positioned restroom fixtures and playground equipment designed for average stature height highlight children's reliance on caregivers and physical differences.

I think that when you're 3, 4, 5, 6 years old and your mom has to boost you up in the restaurant bathroom to wash your hands, it's not that big of a deal. But when you're in fourth, fifth, six, seventh, eighth grade, it's not cool. —Caregiver of a child with HCH

#### 3.2.5 Information Sources & Resource Needs

Children and teens expressed a need for more information on HCH's long‐term impacts and opportunities to connect with peers who share their diagnosis. Caregivers also sought clearer guidance on which specialists to see, potential medications, and likely outcomes, as well as reliable networks for peer support. Many valued the Hypochondroplasia Families Facebook group but also hoped for in‐person meetups centered on HCH. Additional requests included insurance navigation tips and recommendations for adaptive clothing and other consumer products.

##### 3.2.5.1 Focus Groups

Focus group participants highlighted challenges in finding healthcare providers familiar with HCH and expressed a strong desire for more connections with other HCH families. For many, the focus group was their first opportunity to connect with others facing similar experiences, which they found valuable. Some parents noted that while their children participated in events for the dwarfism community, they had mixed feelings about belonging. They emphasized that HCH is a distinct condition, not a mild form of achondroplasia, as it is sometimes portrayed.

This is also my only time in eight years, almost nine years that I've ever spoken with another parent that has a child with HCH. Achondroplasia, yes, but then, you fall in that to where he's too tall for that world, but too small for the average world. So, you're kind of just stuck in the middle. —Parent of a child with HCH

#### 3.2.6 Complications Experienced by People With HCH

##### 3.2.6.1 Focus Groups

**FIGURE 3:**
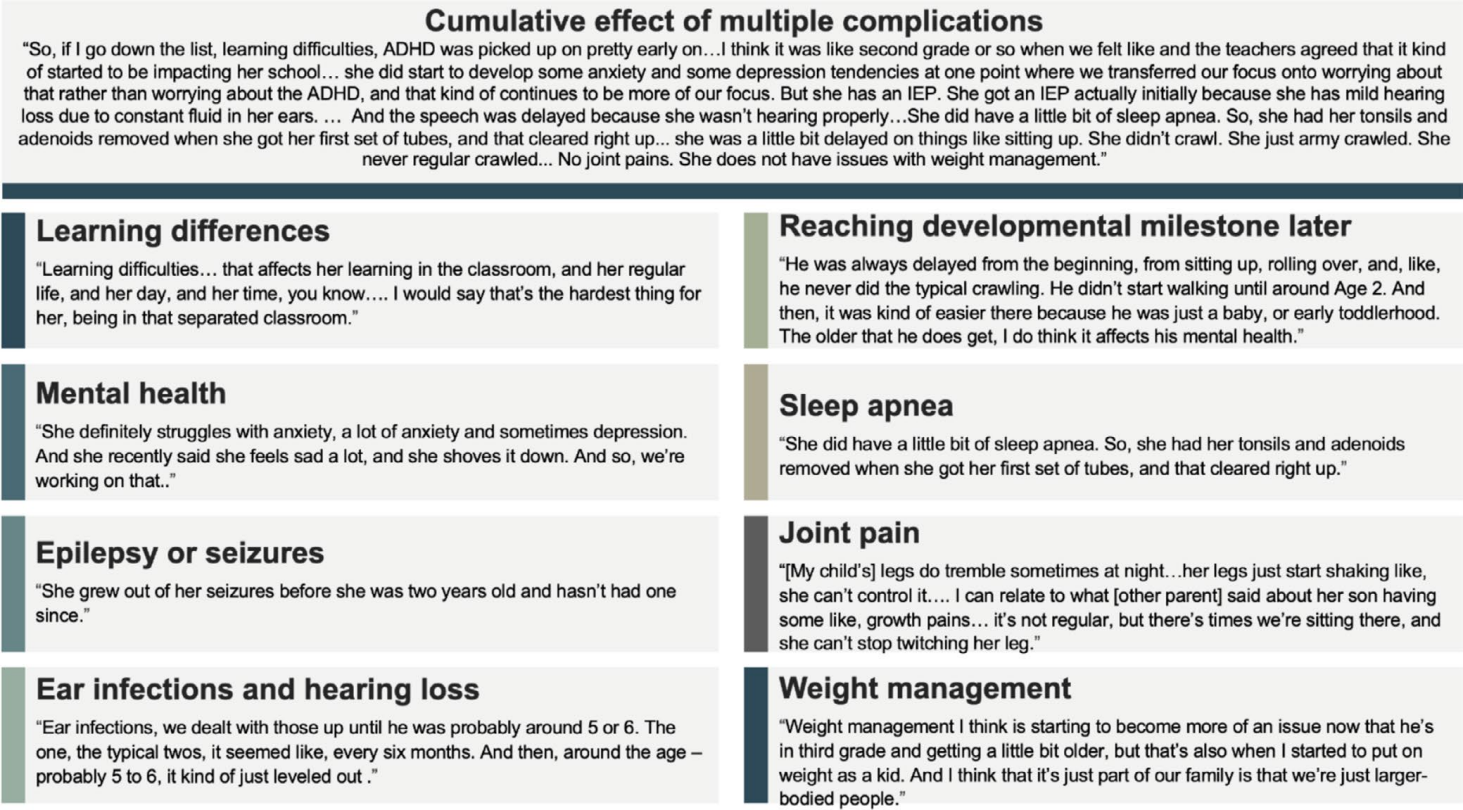
Illustrative quotes of caregiver perspectives on HCH‐related complications.

Focus group participants were asked to reflect on the relative impact of individual complications, with many reporting that the cumulative effect of multiple issues posed the greatest challenge (see Figure 3 for illustrative quotes).

- Learning differences and challenges: Children were often described as bright but easily distracted, with some receiving ADHD, ADD, or autism diagnoses and/or requiring Individualized Education Programs. Separation from same‐age peers sometimes led to isolation and concerns about inequitable resource allocation.
- Developmental delays: Many families noted motor and speech delays, typically addressed through early intervention services (e.g., physical, occupational, and speech therapy). Caregivers differed in whether they attributed these delays directly to HCH or to broader social and environmental factors.
- Mental health concerns: Anxiety was common and often exacerbated by situations that draw attention to physical differences, such as being asked to line up by height. Depression was also reported and often linked to social pressures about height, remarks from others, or feelings of isolation.
- Sleep apnea: Severity ranged from mild to severe. Sleep studies were stressful, but surgical interventions for sleep apnea or Chiari malformation helped some children eliminate apnea episodes. One caregiver described heavy snoring and intermittent pauses in breathing, prompting constant vigilance.
- Epilepsy or seizures: While some children experienced seizures during infancy or early childhood that eventually subsided, families faced significant stress in the meantime, often requiring specialized care and hospital stays. Apneic seizures involving brief cessation of breathing were particularly alarming.
- Joint pain: Periodic joint pain or “growing pains” was common, often affecting knees, ankles, hips, and the lower back. Pain management typically involved over‐the‐counter medications, but more active days could disrupt sleep.
- Ear infections and hearing loss: Recurrent ear infections and mild hearing difficulties were frequent, sometimes requiring multiple sets of ear tubes. Hearing issues occasionally delayed speech and language acquisition, resulting in further therapy needs. Several caregivers ranked these complications among the most difficult aspects of HCH.
- Weight management: Some children struggled to maintain a healthy weight, while others did not. Parents often thought weight challenges were linked to family history rather than HCH. However, one parent remarked that short stature itself contributed to weight challenges.

## 4 Discussion

This study provides valuable insights into the experiences of children with HCH and their caregivers. There are opportunities to address barriers along the diagnostic pathway through additional HCP education. While HCH has a significant impact on daily life, current treatments are invasive. There is an unmet need for new treatments that address short stature and disproportionate limb length, as well as new resources and initiatives to support HCH families and HCPs.

While a 2020 review indicated that HCH is often diagnosed once growth delays become evident in early childhood (Bober et al. 2020), our findings show that most participants in this cohort were diagnosed before their first birthday, aligning with recent reports of prenatal or early‐infant detection (Sabir et al. 2021). Despite earlier diagnoses, families still encountered hurdles, including limited specialist availability and lengthy wait times for appointments and test results. Many healthcare providers remain unfamiliar with HCH, particularly when classic indicators of achondroplasia are absent. There is an unmet need for more training and standardized diagnostic protocols for HCH. Newly developed resources such as an HCH growth chart and a primer on diagnosing skeletal dysplasias are an important starting point (Bober et al. 2020; Legare and Basel 2023; Cheung et al. 2024).

Our study also highlights the unmet need for therapies aimed at height gain or improved limb proportionality. About half of the families interviewed had explored either limb‐lengthening surgery, growth hormone therapy, or both. Limb‐lengthening was widely seen as beneficial for enhancing overall height. This is a relatively high proportion and may indicate a more severe cohort. However, undergoing the surgery demands substantial commitment from families and also involves significant pain for children. Experiences with growth hormone therapy were mixed, with parents unsure whether it was effective in increasing height. These observations are consistent with prior evidence showing variable responses to growth hormone in HCH (Pinto et al. 2024; Massart et al. 2015). Notably, some families opted out of invasive treatments altogether, concentrating instead on mental health and adaptive strategies when other serious comorbidities were not present.

Children and caregivers reported a range of health complications often overlapping: developmental delays, sleep apnea, and recurrent ear infections were common, reflecting broader patterns in skeletal dysplasias (Bober et al. 2020). This study provides additional insights into how multiple challenges—learning differences, anxiety or depression, chronic joint pain—interact to shape children's overall well‐being. These emotional and mental health challenges echo findings from other rare skeletal conditions (Billich et al. 2023). They suggest that mental health screening should be considered among those with HCH.

Finally, this study stresses the importance of social support and access to reliable information about HCH. Several topics of interest, including the natural history of HCH, are covered in a brief summary available on the Little People of America website (Pauli 2009). Online platforms such as the Hypochondroplasia Families Facebook group offer peer‐to‐peer support and information exchange, while annual conferences—such as those hosted by The Chandler Project—bring together patients, researchers, and medical professionals, with a dedicated HCH‐focused meeting held in 2024 (Achondroplasia and Skeletal Dysplasia Research Conference 2024). Healthcare providers can enhance support for families affected by HCH by directing them to these resources and opportunities for community engagement. However, participants still reported a desire for more practical guidance—such as tips for navigating insurance or finding adaptive clothing—that can be disseminated through healthcare providers or patient advocacy organizations.

### 4.1 Limitations

This research has several key strengths. We included a cohort of people with a physician‐confirmed diagnosis rather than relying on self‐reported HCH. All patient‐facing materials were reviewed and edited by a caregiver partner and child with HCH, ensuring clarity and relevance. However, the study findings must also be considered in light of limitations. Participants described their past experiences, which may be subject to recall bias. Our sample included primarily individuals diagnosed with HCH within their first year of life, which differs from other estimates in the literature. Thus, their experiences may not be generalizable to all people with HCH. Additionally, participants were recruited from HCP offices and online patient communities. Almost 25% of the children represented in the cohort had undergone limb‐lengthening surgery, indicating that participants may be a more severe or interventionally prone cohort. Participants of online patient communities may be more activated than other patients.

## 5 Conclusion

This study addresses the limited understanding of people's experiences living with HCH. We found that diagnostic pathways vary significantly, with signs of HCH identified in utero or during infancy or early childhood. Families described complex psychosocial burdens that include impacts on daily activities and emotional challenges due to height differences and disproportionate limb length. Additionally, many people with HCH have complications that go beyond short stature and include developmental delays, learning differences, and seizures. Families desire more support and resources related to HCH. Future efforts should focus on holistic, patient‐centered strategies to better support individuals with HCH and their families.

## Author Contributions

Elisabeth M. Oehrlein, Reni Pekala, Stacie Cavallaro, Margaret Cho, and Emily S. Reese conceptualized the study. Elisabeth M. Oehrlein, Emily S. Reese, Ankita Saxena, and Joe Vandigo collected and analyzed the data. All authors contributed to the interpretation of the results and preparation of the manuscript.

## Conflicts of Interest

E.M.O., A.S., and J.V. are employees of Applied Patient Experience LLC (AppliedPX), a company that contracts with various nonprofit organizations, pharmaceutical companies, and academic institutions. They received salary support from BioMarin to conduct the research described in this manuscript. E.S.R. was employed by AppliedPX while the research was conducted and is now an employee of Otsuka Pharmaceuticals. R.P. is a volunteer for the Hypochondroplasia Families Community. She received an honorarium for advising AppliedPX on this study. S.C. and M.C. are employees and shareholders of BioMarin Pharmaceuticals. C.C. is the Founder of The Chandler Project, which has received conference support from BioMarin and is member of advisory boards for QED Therapeutics, a BridgeBio company, and Ascendis Pharma. A.D. served as a consultant for BioMarin, but all compensation has been paid to Children's National Hospital and he has not received any personal compensation from BioMarin.

## Supporting information


**Supporting Information S1:** mgg370151‐sup‐0001‐Supinfo.docx.

## Data Availability

The data that support the findings of this study may be available on request from the corresponding author. The data are not publicly available due to privacy or ethical restrictions.
