# Supplementary material for: Living With Hypochondroplasia: A Qualitative Exploration of Children's and Caregivers' Experiences, Challenges, and Unmet Needs
Source: Mol Genet Genomic Med. 2025 Nov 14;13(11):e70151. doi: 10.1002/mgg3.70151 (PMC12617553; doi:10.1002/mgg3.70151)
Supplement: Supplementary file 1 — Supporting Information S1: mgg370151‐sup‐0001‐Supinfo.docx. [file MGG3-13-e70151-s001.docx]

**Supplemental Materials** 1. Example Interview and Focus Group Questions

Example Interview Questions (Caregivers)

- Could you please tell me more about your child’s experience finding out that he or she has hypochondroplasia?
- Which providers did they see along the journey to finding out about hypochondroplasia?
- Did your child have any tests, such as genetic testing, an X-ray or other lab tests?
- Please tell me more about complications associated with your child’s hypochondroplasia. This could be changes to how their body looks or how they feel or move. What have you noticed? How have they changed over time? How do the clinical features and/ or complications impact your child’s day-to-day life?
- Does your child have any trouble thinking, concentrating, expressing thoughts, or solving problems? Please tell me what that is like.
- What questions do you have about hypochondroplasia?

Example Interview Questions (Child)

- How did you learn that you have hypochondroplasia?
- Do you avoid certain sports or physical activities because of hypochondroplasia? Please describe what you avoid and why?
- Please think about hobbies, school, and other day-to-day activities that are important to you or that you like to do. How does hypochondroplasia impact your ability or experience doing those things?
- What questions do you have about hypochondroplasia?

Example Focus Group Questions

- During the first phase of this project, we heard about people’s experiences with height or limb length differences relative to average stature individuals, but we also heard about other complications of HCH. I will put them up on a slide.

| **[Slide]**   - Learning difficulties or trouble thinking, concentrating, or expressing thoughts or solving problems. - Reaching developmental milestone later than their peers, for example not talking, walking, running at the same time as other children their age. - Mental health, including anxiety and depression - Sleep apnea, a sleep disorder that causes breathing to stop and start, over and over - Epilepsy or seizures - Joint pain - Ear infections - Hearing loss - Weight management - Others? |
| --- |

- Has your child experienced any of these complications, or other complications, of HCH? If so, could you please describe them for me?
- How do complications of HCH affect your child’s daily life? For example, what impacts your child’s school, routine, social life, relationships, or health care?

Supplemental Table 1. Focus group participant and child characteristics

| **Characteristic** | **n** | **(%)** |
| --- | --- | --- |
|  |  |  |
| **Caregiver sex** |  |  |
| Female | 9 | 90% |
| Male | 1 | 10% |
|  |  |  |
| **Child age today, years** |  |  |
| Mean, SD |  | 10.7 (4.67) |
| Median, Min - Max |  | 10.0 (5 – 19) |
|  |  |  |
| **Child height at focus group, inches** |  |  |
| Mean, SD |  | 45.4 (6.75) |
| Median, Min – Max |  | 45.5 (34 – 55) |
|  |  |  |
| **Diagnosed at birth** |  |  |
| Yes | 3 | 30% |
| No | 7 | 70% |
|  |  |  |
| **Diagnosis age if not at birth, years** |  |  |
| Mean, SD |  | 0.93 (0.89) |
| Median, Min – Max |  | 1.5 (0 – 2) |
|  |  |  |
| **Sex at birth** |  |  |
| Female | 4 | 40% |
| Male | 6 | 60% |
|  |  |  |
| **Ethnicity** |  |  |
| Hispanic | 1 | 10% |
| Non-Hispanic | 9 | 90% |
|  |  |  |
| **Race** |  |  |
| American Indian or Alaska Native | 0 | - |
| Asian | 0 | - |
| African American | 0 | - |
| Native Hawaiian or Other Pacific Islander | 0 | - |
| White | 8 | 80% |
| Two or More Races | 2 | 20% |
| Other | 0 | - |
|  |  |  |
| **Community setting** |  |  |
| Rural | 3 | 30% |
| Suburban | 7 | 70% |
| Urban | 0 | - |
|  |  |  |
| **Census region** |  |  |
| Midwest | 4 | 40% |
| Northeast | - | - |
| South | 5 | 50% |
| West | 1 | 10% |
|  |  |  |
| **Household income** |  |  |
| <$25,000 | 1 | 10% |
| $25,000-49,000 | 0 | - |
| $50,000-99,000 | 3 | 30% |
| $100,000-149,000 | 4 | 40% |
| ≥$150,000 | 2 | 20% |
|  |  |  |
| **Complications and comorbidities^1^** |  |  |
| Shorter than Peers | 10 | 100% |
| Shorter Legs and/or Arms | 10 | 100$ |
| Enlarged Head Size | 8 | 80% |
| Bowlegs | 5 | 50% |
| Learning Difficulties/ Cognitive Impairment^2^ | 5 | 50% |
| Limited Elbow Extension | 7 | 70% |
| Recurrent Ear Infection | 6 | 60% |
| Motor Development Delays | 4 | 40% |
| Obstructive Sleep Apnea | 5 | 50% |
| Pain (joints, back, or neck) | 4 | 40% |
| Epilepsy or Other Seizure Disorder | 3 | 30% |
| Anomalies on Brain MRI | 3 | 30% |
| Hearing Loss | 2 | 20% |
| Lumbar Lordosis | 3 | 30% |
| Central Sleep Apnea | 2 | 20% |
| Kyphosis (rounding of the upper back) | 1 | 10% |
| Scoliosis (spinal curve to the side) | 1 | 10% |
| Hydrocephalus |  | - |
| Neuropathy |  | - |
|  |  |  |
| **HCH Treatments Received (if any)** |  |  |
| Growth Hormone Therapy | 0 | - |
| Limb Lengthening Surgery | 1 | 10% |

^1^Participants were able to select no or multiple complications and comorbidities, or treatments

^2^For example: language delay, ADHD, autism, trouble concentrating
